# An analysis of factors and pathways related to rehabilitation motivation in stroke patients based on self-determination theory

**DOI:** 10.3389/fneur.2026.1789949

**Published:** 2026-04-21

**Authors:** Xuan Wang, Yanfei Hu, Rui Wang, Xiaolu Wei, Qian Shao, Qian Su

**Affiliations:** 1 School of Nursing, Lanzhou University, Lanzhou, China; 2 Department of Rehabilitation Medicine, The First Hospital of Lanzhou University, Lanzhou, China; 3 Spine Surgery Department, Gansu Provincial Hospital, Lanzhou, China

**Keywords:** Rehabilitation motivation, self-determination theory, self-efficacy, social support, stroke

## Abstract

**Objectives:**

Based on self-determination theory, this study investigates the current state of rehabilitation motivation among stroke patients and its psychosocial correlates, providing evidence for developing a motivation-enhancing intervention model.

**Methods:**

Using convenience sampling, 430 stroke patients were recruited from the inpatient wards of the neurology, neurosurgery, and rehabilitation medicine departments at two Grade A Level 3 hospitals in Lanzhou. Data collection employed a demographic questionnaire, Stroke Rehabilitation Motivation Scale, Social Support Rating Scale, General Self-Efficacy Scale, Hospital Anxiety and Depression Scale, and Craig Hospital Inventory of Environmental Factors. Descriptive analysis, correlation analysis, and multiple linear regression analysis were performed using SPSS 26.0, while structural equation modeling was constructed with AMOS 28.0.

**Results:**

The mean rehabilitation motivation score for stroke patients was (105.73 ± 22.03) points, indicating a moderately high level. Multivariate linear regression analysis revealed that monthly household income, family relationships, employment status, rehabilitation duration, self-efficacy, social support, anxiety-depression, and environmental factors were significant predictors of rehabilitation motivation (*p* < 0.05), explaining 70.4% of total variance. The results of the structural equation modeling indicated that social support (*β* = 0.17, *p* = 0.002) and self-efficacy (*β* = 0.132, *p* < 0.001) showed a direct positive association with the rehabilitation motivation of stroke patients, while anxiety and depression (*β* = −0.285, *p* < 0.001) and environmental factors (*β* = −0.496, *p* < 0.001) showed a direct negative association with the level of rehabilitation motivation among stroke patients, while self-efficacy and anxiety and depression demonstrated a significant indirect effect in the relationship between social support and environmental factors, consistent with a mediational role.

**Conclusion:**

Rehabilitation motivation in stroke patients is influenced by multifaceted factors. It is recommended to implement family-centered, psychosocially integrated motivational intervention strategies, incorporating digital technology to enhance the rehabilitation experience and optimize the rehabilitation support system.

## 1 Introduction

Stroke has become one of the leading causes of long-term disability worldwide, with its disease burden intensifying against the backdrop of China’s accelerating population ageing (1). Post-stroke functional impairment not only severely compromises patients’ physical and psychological well-being but also imposes a heavy burden on families and society (2). Rehabilitation therapy, as a key pathway to restoring function and enhancing quality of life, relies heavily on the patient’s active participation. A multicenter randomized controlled clinical trial on early rehabilitation for hemorrhagic stroke demonstrated that initiating rehabilitation within 48 h of intracerebral hemorrhage improves survival rates and enhances functional outcomes at 6 months post-onset, including quality of life and anxiety levels (3). However, the widespread issue of insufficient rehabilitation motivation in clinical practice severely constrains the rehabilitation process and outcomes.

Motivation is the intrinsic driving force that determines individual behavior. Self-determination theory’s primary assumption is the emphasis on three basic psychological needs: relatedness, competence, and autonomy. It suggests that meeting these requirements is essential to boosting behavioral willingness and volition, which in turn determines the degree of self-determination in one’s behaviors. This theory clarifies how human cognitive and personality development is influenced by a dialectical interaction between external social surroundings and internal psychological elements. Therefore, this study posits that rehabilitation motivation constitutes “the intrinsic driving forces (such as self-efficacy and sense of accomplishment) exhibited by individuals during the rehabilitation process, coupled with their tendency towards reactive behaviors in response to the external environment (such as social support and rehabilitation resources)”.

In recent years, with the deepening development of the“bio-psycho-social” medical model, the role of psychological factors in stroke rehabilitation has garnered increasing attention. Rehabilitation motivation, as the core psychological driver linking rehabilitation intent and behavior, is a significant factor influencing patients’ rehabilitation engagement (Observable rehabilitation participation behaviors) and functional recovery (4). According to research (5), rehabilitation motivation may lower mortality by increasing treatment adherence, despite having little impact on the recovery of cognitive and motor functions. This underscores the independent importance of motivation.

However, research indicates that over half of stroke patients exhibit insufficient autonomy and engagement during rehabilitation (6). Due to a lack of awareness about rehabilitation and insufficient motivation, many individuals fail to undertake timely and effective rehabilitation exercises, thereby missing the optimal recovery window (7, 8). Previous stroke rehabilitation studies have predominantly focused on objective indicators such as physiological function, with insufficient systematic exploration of rehabilitation motivation as a key subjective factor. In clinical practice, a “motivation crisis” stemming from disease characteristics is prevalent, severely impeding the rehabilitation process. Although some studies have begun to examine factors influencing rehabilitation motivation, these often remain confined to single-level analyses, lacking systematic integration grounded in theoretical frameworks. Particularly, exploration of the underlying psychosocial mechanisms remains superficial. Self-Determination Theory emphasizes that the fulfillment of an individual’s intrinsic psychological needs is central to sustaining long-term behavioral motivation (9).

Given this, the goal of the current study is to systematically assess stroke patients’ rehabilitation motivation within the framework of self-determination theory, analyzing its correlates and pathways. The goal of this project is to create an evidence-based framework for applying focused, methodical motivational treatments in clinical settings.

## 2 Materials and methods

### 2.1 Study design and participants

In order to examine the factors impacting stroke patients’ rehabilitation motivation and their underlying mechanisms, this cross-sectional study examined adult stroke patients at Lanzhou University First Hospital and Gansu Provincial Hospital. Convenience sampling was used in this study to select individuals between [December 2024] and [August 2025] from two Grade A tertiary hospitals in Northwest China. Both hospitals are major rehabilitation centers serving urban and rural populations in the region. Each participant was recruited from the inpatient wards of these two hospitals’ departments of neurology, neurosurgery, and rehabilitation medicine. The following was the recruitment procedure: Every week, newly admitted patients who met the inclusion criteria were screened by the research team. The attending physician or research nurse informed participants and their families of the study’s goals following preliminary selection through electronic medical records. Once the patient’s condition stabilized (usually 48–72 h following admission), researchers performed in-person interviews with prospective patients to ensure final eligibility and acquire written informed consent.

The inclusion criteria were as follows: (1) patients diagnosed with stroke (10); (2) Age ≥ 18 years; (3) conscious, with stable vital signs, and able to complete the questionnaire independently or with the investigator’s assistance. Exclusion criteria included the following: (1) Individuals with severe cardiac, hepatic, or pulmonary complications, malignant tumors, hematological disorders, or pre-stroke disabilities; (2) patients with transient ischemic attacks; (3) diagnosed with cognitive impairment (Mini-Mental State Examination: illiterate ≤17 points, primary education ≤20 points, secondary education and above ≤24 points), with a history of dementia and other psychiatric conditions. Paper-based questionnaires were used for data collection, 430 participants made up the study’s final sample size, patients from the rehabilitation department and the neurology/neurosurgery departments accounted for a ratio of 4 to 1. The questionnaire asked questions about demographics (gender, age, marital status, level of education, etc.), diseases (stroke type, length of illness, Activities of Daily Living (ADL) scores, etc.), and caregivers (caregiver type). For this study, 442 questionnaires were mailed out and collected; 430 valid replies were received, yielding an effective response rate of 97.2%.

### 2.2 Ethical considerations

This study has been approved by the Ethics Committee of Lanzhou University (LZUHLXY20240079). Each participant provided informed consent, paper-based, before participation, and was informed of the study’s objectives and voluntary nature. Respondents could withdraw at any time.

### 2.3 Theoretical framework

**Figure 1:**
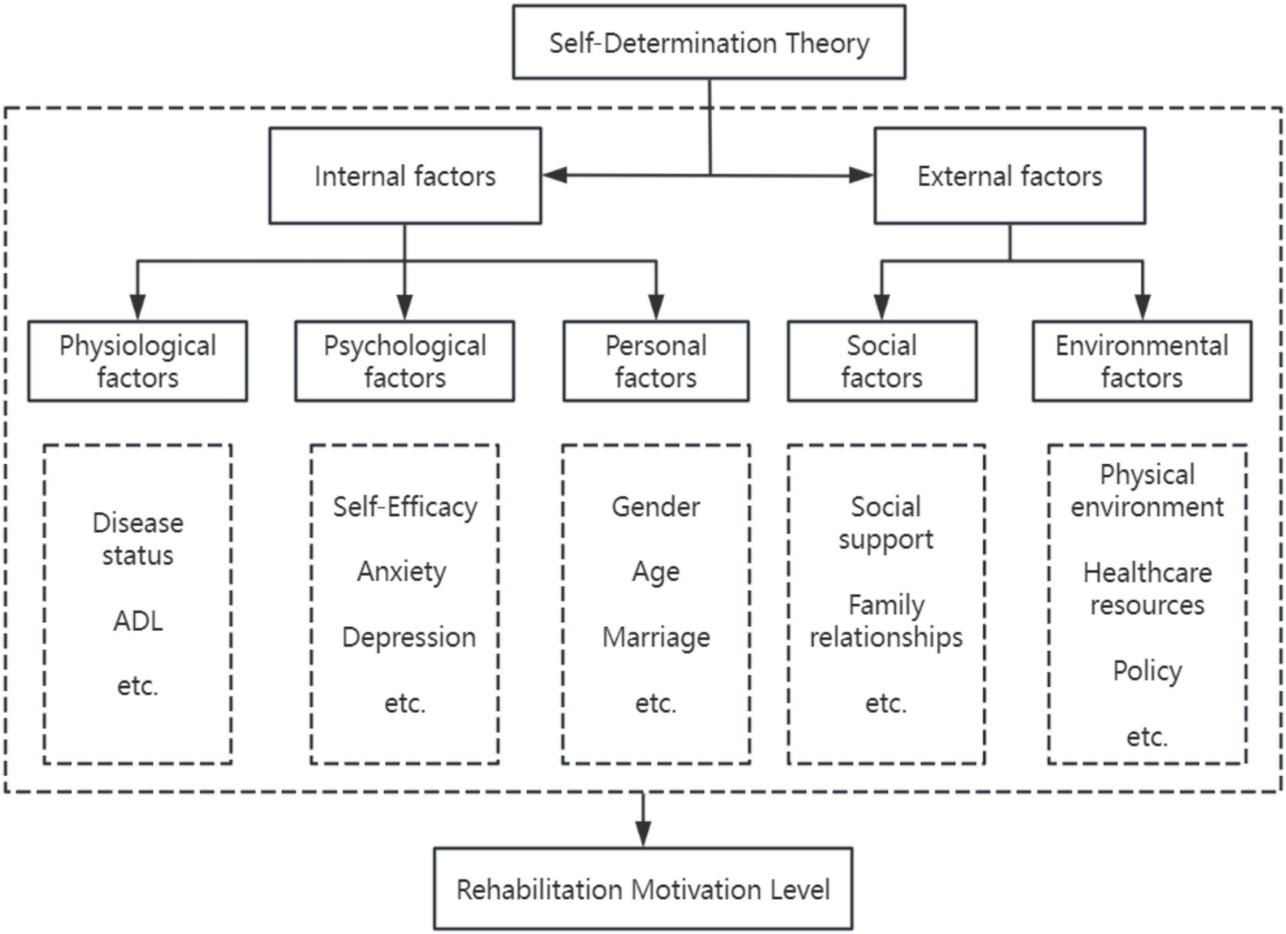
Theoretical framework based on self-determination theory.

Based on self-determination theory, this study develops a theoretical framework for variables affecting stroke patients’ rehabilitation motivation (Figure 1). It investigates how rehabilitation motivation in stroke patients was associated with physiological factors (disease status and functional capacity), psychological factors (self-efficacy and emotional regulation), personal factors (demographic characteristics), social factors (social support, family relationships, and caregivers), and environmental factors (physical environment, healthcare resources, and healthcare policies).

Based on this theoretical framework, we map the selected core variables to the SDT structure as follows:

1. Self-efficacy was conceptualized as an indicator of the competence need. According to SDT, experiences of effectiveness and mastery (closely aligned with self-efficacy beliefs) directly nourish the competence need, thereby enhancing intrinsic motivation.
2. Social support was conceptualized as a key source of relatedness satisfaction. Emotional support, encouragement, and instrumental assistance from family, friends, and healthcare providers fulfill patients’ need for connection and belonging, which in turn facilitates motivation internalization.
3. Anxiety and depression were conceptualized as manifestations of basic psychological need frustration. Negative emotional states undermine intrinsic motivation.
4. Environmental factors (physical environment, attitudes and support, services, and policies) were conceptualized as autonomy support or hindrance factors. SDT emphasizes that social-contextual conditions that support autonomy facilitate motivation, while controlling or restrictive environments undermine it.

**Figure 2:**
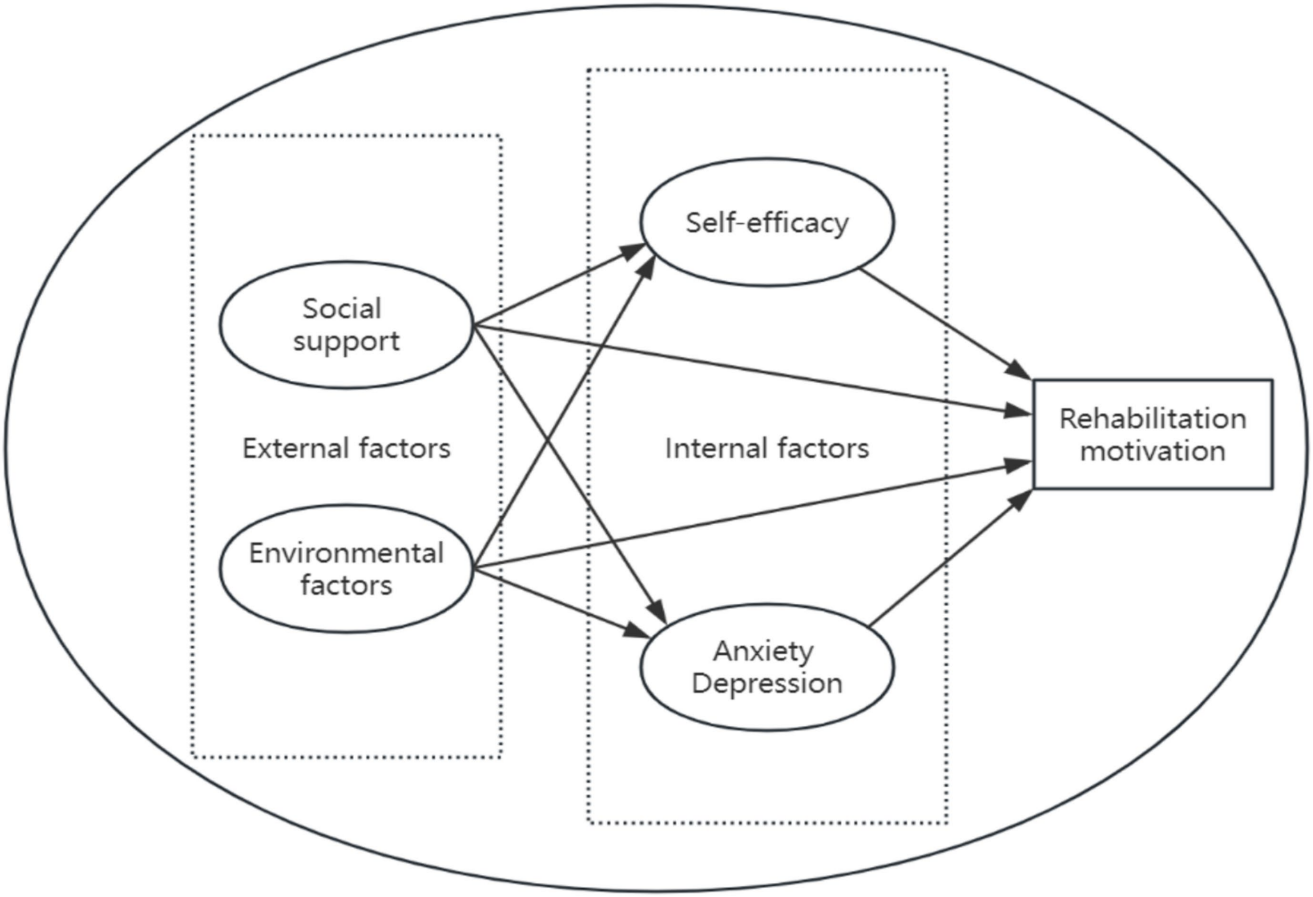
Schematic diagram of the hypothesis model for factors influencing rehabilitation motivation in stroke patients.

Self-determination theory posits that individuals tend to internalize external rules, develop autonomous motivation, and show more initiative, willpower, and tenacity when the external environment supports and satisfies these demands. On the other hand, regulated motivation develops when requirements are not met, which reduces behavioral autonomy. The research hypothesis model diagram for this study has been formulated (Figure 2).

### 2.4 Measures

#### 2.4.1 The stroke rehabilitation motivation scale

The SRMS, based on self-determination theory, was developed to assess the rehabilitation motivation of stroke patients and is capable of systematically and scientifically evaluating their level of rehabilitation motivation (11). The scale’s 28 items span seven dimensions (amotivation, extrinsic motivation identification, extrinsic motivation regulation, extrinsic motivation introjected, intrinsic motivation knowledge, intrinsic motivation stimulation, and intrinsic motivation accomplishment). Higher overall scores on a five-point Likert scale (1 = Strongly disagree, 5 = Strongly agree) signify more patient rehabilitation motivation. A sample of 429 stroke patients receiving rehabilitation care in Qingdao, China, was used to translate and validate the Chinese version of the SRMS (12). The SRMS in Chinese has a Cronbach’s alpha value of 0.851 and a test–retest reliability of 0.922.

#### 2.4.2 General self-efficacy scale

This scale measures an individual’s general, rather stable sense of self-efficacy in managing stressful situations. It employs ten items spanning a single dimension on a four-point Likert scale (1 = Completely incorrect, 5 = Completely correct), greater scores correspond to higher levels of self-efficacy. The Chinese version of the GSES has an internal consistency alpha value of 0.87 and a test–retest reliability coefficient of 0.83, it is widely used among chronic disease patients (13). This scale has demonstrated good psychometric properties in stroke patients (14).

#### 2.4.3 Social support rating scale

This scale is one of the most widely used social support assessment tools in the fields of rehabilitation and psychology in China. Ten items total, divided into three categories (subjective support, objective support, and utilization of support), make up this scale (15). A low level is indicated by ≤22 points, a medium level by 23–44 points, and a high level by 45–66 points. The score range is 12–66 points. Greater social support is indicated by higher scores. The scale has a test–retest reliability value of 0.920 and Cronbach’s alpha value between 0.89 and 0.94. Previous studies have confirmed the feasibility and applicability of this scale in stroke patients (16).

#### 2.4.4 Hospital anxiety and depression scale

This measure was created by Zigmond and Snaith to check hospitalized patients for signs of depression and anxiety (17). It consists of 14 items in two categories (depression and anxiety). Items related to somatization symptoms are not included in this measure. Higher scores on a 4-point Likert scale (0–3) denote more severe depression or anxiety. The Cronbach’s alpha value for the Chinese versions of the anxiety subscale, depression subscale, and overall scale were 0.904, 0.869, and 0.807, respectively. This scale has been widely applied in stroke populations (18).

2.4.5. Craig hospital inventory of environmental factors (CHIEF).

This scale was designed by Whiteneck et al. (19) and then translated into Chinese by Liao et al. (20). Stroke populations have validated the Chinese version of CHIEF (21). The physical environment, attitudes and support, work and study, services and assistance, and policies are the five dimensions that comprise its 12 items. Each item’s score is calculated as follows: frequency score (0–4 points) × severity score (0–2 points). More severe perceived environmental constraints are indicated by a higher total score. With a test–retest reliability coefficient of 0.80 and a Cronbach’s alpha value of 0.889, the Chinese version of CHIEF exhibits outstanding reliability. Additionally, the measure demonstrates good discriminant and convergent validity.

### 2.5 Statistical analysis

Excel was implemented to enter the data, and the SPSS 26.0 software was used for statistical analysis. Measurement data were expressed as mean ± standard deviation (M ± SD), whereas count data were described as frequencies and percentages. To look into influential factors, multiple linear regression analysis, Pearson correlation analysis, one-way analysis of variance, and independent samples *t*-tests were used. AMOS Version 28.0 was used to create structural equation modeling in order to investigate path linkages between variables. Statistical significance was defined as a *p*-value of less than 0.05.

## 3 Results

### 3.1 General demographic characteristics and variance analysis or *t*-tests of rehabilitation motivation among stroke patients

The mean age of 430 stroke patients, who ranged in age from 18 to 82, was (55.81 ± 14.7) years. Of the group, 66% were men. For more information, see Table 1.

**Table 1:** Univariate analysis of factors associated with rehabilitation motivation in stroke patients.

Variables	Group	*n* = 430	SRMS score (M ± SD)	*t*/*F*	*P*	LSD
Gender				0.077	0.836	
	Male	284	105.89 ± 21.96			
	Female	146	105.42 ± 22.25			
Age (years)				4.636	0.01	
	18–45^a^	96	110.69 ± 17.58			
	46–59^b^	146	106.62 ± 21.15			
	≥60^c^	188	102.51 ± 24.20			
Marriage				2.804	0.04	a > c,d
	Unmarried^a^	39	110.59 ± 21.19			
	Married^b^	309	116.68 ± 20.81			
	Divorced^c^	31	99.35 ± 27.28			
	Widowed^d^	51	100.18 ± 25.05			
Level of education				5.027	0.002	a < c,d; b < d
	Primary and below^a^	96	100.21 ± 26.64			
	Middle school^b^	126	103.69 ± 22.77			
	High school^c^	98	107.6 ± 20.32			
	College and above^d^	110	111.23 ± 16.27			
Work				4.348	0.014	a > c
	In post^a^	96	111.33 ± 19.66			
	Retirement^b^	106	105.46 ± 22.16			
	Unemployed^c^	228	103.5 ± 22.58			
Residence				−0.294	0.769	
	Town	259	105.48 ± 22.39			
	Village	171	106.12 ± 21.53			
Family monthly income (RMB/mouth)				59.98	0.000	a < b < c
	<2,000^a^	27	79.33 ± 31.33			
	2,000–5,000^b^	255	101.43 ± 22.46			
	>5,000^c^	148	117.96 ± 7.77			
Ways of paying medical expenses				0.882	0.415	
	Health insurance	389	105.42 ± 22.54			
	Public expense	7	116 ± 5.83			
	Self pay	34	107.26 ± 17.32			
Family relationships				32.225	0.000	a < b < c
	Generally^a^	39	92.33 ± 29.53			
	Good^b^	225	100.99 ± 22.97			
	Excellent^c^	166	115.31 ± 13.39			
Level of disease understanding				12.095	0.000	a < b < c,d
	Not^a^	98	97.32 ± 25.77			
	Generally^b^	160	104.2 ± 22.51			
	Relatively^c^	123	111.93 ± 16.76			
	Very^d^	49	118.5 ± 7.21			
Rehabilitation period (mouth)				16.523	0.000	a < b,c,d
	<1^a^	251	99.81 ± 24.57			
	1–3^b^	96	112.4 ± 16.24			
	3–6^c^	41	115.51 ± 14.95			
	>6^d^	42	116.33 ± 6.23			
Types of stroke				0.025	0.98	
	Ischemic	216	105.76 ± 22.92			
	Haemorrhagic	214	105.71 ± 21.15			
Number of strokes				−0.1	0.92	
	1	385	105.70 ± 22.32			
	≥2	45	106.04 ± 19.55			
Duration of illness (mouth)				9.145	0.000	a < c,d; b < c,d
	<1^a^	177	103.36 ± 22.76			
	1–3^b^	147	101.86 ± 25.08			
	3–6^c^	53	113.94 ± 14.04			
	>6^d^	53	116.21 ± 6.70			
Paralysed limbs				0.526	0.591	
	Unilateral	363	105.98 ± 21.89			
	Bilateral	31	101.87 ± 25.47			
	None	36	106.61 ± 20.52			
Are there any complications?				0.049	0.961	
	Yes	344	105.76 ± 22.08			
	No	86	105.63 ± 21.97			
Whether to retain the catheter				−2.591	0.011	
	Yes	83	99.06 ± 27.326			
	No	347	107.33 ± 20.29			
ADL score				15.024	0.000	a < b < c,d,e
	≤20^a^	61	89.41 ± 27.01			
	21–40^b^	70	100.41 ± 26.00			
	41–60^c^	91	107.56 ± 18.86			
	61–99^d^	129	110.55 ± 17.58			
	100^e^	79	113.08 ± 16.18			
Caregiver				3.09	0.016	a,b,d < e
	Spouse^a^	120	104.55 ± 21.48			
	Children^b^	117	102.82 ± 23.26			
	Parents^c^	63	109.63 ± 18.68			
	Other^d^	103	104.96 ± 23.50			
	None^e^	27	117.44 ± 16.04			

SRMS: stroke rehabilitation motivation scale; ADL: activities of daily living; M: mean; SD: standard deviation; a-e denote different groups of variables; LSD, Least Significant Difference (LSD) *post-hoc* test, *p* < 0.05).

### 3.2 Descriptive statistics and correlations of variables

The mean value and standard deviation (M ± SD) for each variable were as follows: SRMS (105.73 ± 22.03). 39 (9.1%), 128 (29.8%), and 263 (61.2%) patients had low, moderate, and high levels of motivation, respectively. With the highest score (15.32 ± 4.10) for the intrinsic motivation dimension, patients’ motivation to heal has begun to become more internalized. This is an indicator of a psychologically positive prognosis. GSES (29.74 ± 6.82), SSRS (39.39 ± 10.15), Anxiety (7.24 ± 5.21), Depression (7.26 ± 5.48), and CHIEF (16.66 ± 17.02).

Rehabilitation motivation showed significant negative correlations with anxiety (*r* = −0.468, *p* < 0.001), depression (*r* = −0.576, *p* < 0.001), and environmental barriers (*r* = −0.694, *p* < 0.001), but significant positive correlations with self-efficacy (*r* = 0.567, *p* < 0.001) and social support (*r* = 0.525, *p* < 0.001). See Table 2.

**Table 2:** Correlations between rehabilitation motivation and self-efficacy, social support, anxiety and depression, and environment.

Variable	Rehabilitation motivation	Self-efficacy	Social support	Anxiety	Depression	Environment
Rehabilitation motivation	1					
Self-efficacy	0.567**	1				
Social support	0.525**	0.485**	1			
Anxiety	−0.468**	−0.384**	−0.267**	1		
Depression	−0.576**	−0.564**	−0.454**	0.589**	1	
Environment	−0.694**	−0.429**	−0.435**	0.306**	0.401**	1

**Significant correlation at the 0.01 level (two-tailed).

### 3.3 Multivariate analysis of factors associated with rehabilitation motivation in stroke patients

Rehabilitation motivation was the dependent variable in a multivariate linear regression analysis that included self-efficacy, social support, anxiety, depression, and environmental factors as independent variables in addition to statistically significant variables from the one-way ANOVA. The findings showed that among stroke patients, age, monthly household income, family relationships, length of rehabilitation, self-efficacy, degree of social support, anxiety and depression, and environmental factors were significant predictors of rehabilitation motivation (*p* < 0.05), accounting for 70.4% of the variance (see Table 3).

**Table 3:** Results of multivariate linear regression analysis of factors associated with rehabilitation motivation in stroke patients.

Variable	Coefficient of regression		Standardized regression coefficient	*t*	*P*	Collinearity statistics	
	B	SE	*β*			Tolerance	VIF
(Constant)	59.04	7.095	–	8.322	0	–	–
Retirement	−4.06	1.946	−0.08	−2.086	0.038	0.474	2.107
Family monthly income	6.342	1.219	0.165	5.203	<0.001	0.686	1.459
Family relationships	3.527	1.054	0.1	3.346	0.001	0.772	1.296
Rehabilitation period	3.001	1.281	0.135	2.343	0.02	0.207	4.837
Self-efficacy	0.434	0.114	0.135	3.817	<0.001	0.555	1.803
Social support	0.264	0.073	0.122	3.603	<0.001	0.605	1.653
Anxiety	−0.724	0.148	−0.171	−4.882	<0.001	0.56	1.786
Depression	−0.365	0.161	−0.091	−2.265	0.024	0.43	2.324
Environmental factors	−0.497	0.042	−0.384	−11.825	<0.001	0.653	1.532

SE: standard error; VIF: variance inflation factor; *F* = 41.836, *p* < 0.001; *R*^2^ = 0.721, adjusted *R*^2^ = 0.704.

While individual bivariate correlations between predictors and rehabilitation motivation were moderate in magnitude (*r* ranging from −0.694 to 0.567), their collective contribution to explaining variance in the multivariate model (70.4%) reflects the cumulative and complementary effects of multiple psychosocial and environmental factors.

### 3.4 Structural equation model of rehabilitation motivation in stroke patients

#### 3.4.1 Model fitting and adjustment

Thirteen factors with eigenvalues greater than one were identified by Harman’s one-factor test, with the first component accounting for 35.38% of the variance. This suggests that there is no significant common method bias in the research. Internal factors were identified as mediating variables using the theoretical framework of self-determination theory and the results of multiple linear regression and correlation analysis. AMOS 28.0 was used to build a structural equation model. We performed a Bootstrap analysis using 5,000 resamples in light of this strong model fit to guarantee the validity and dependability of the statistical findings. *χ*^2^/df = 3.462, RMSEA = 0.076, CFI = 0.933, TLI = 0.919, and IFI = 0.933, NFI = 0.908, and SRMR = 0.18 are the initial model fit results.

The initial model had an SRMR of 0.18, indicating poor fit. Among the environmental factors, both the attitude/support and social support variables aimed to measure patients’ perceived external support. According to SDT, a supportive environment is a prerequisite for fulfilling basic psychological needs. Social support directly satisfies the need for relatedness while also serving as a vital resource for individuals to maintain autonomy and competence in adverse environments. When the environment is supportive, social support is more effective; when social support is abundant, patients are more motivated to utilize environmental resources. Theoretically, these two variables exhibit a synergistic covariate relationship. Therefore, based on the adjustment index and incorporating Self-Determination Theory, the covariance parameter between them was released to allow for their correlation in the model revision.

**Figure 3:**
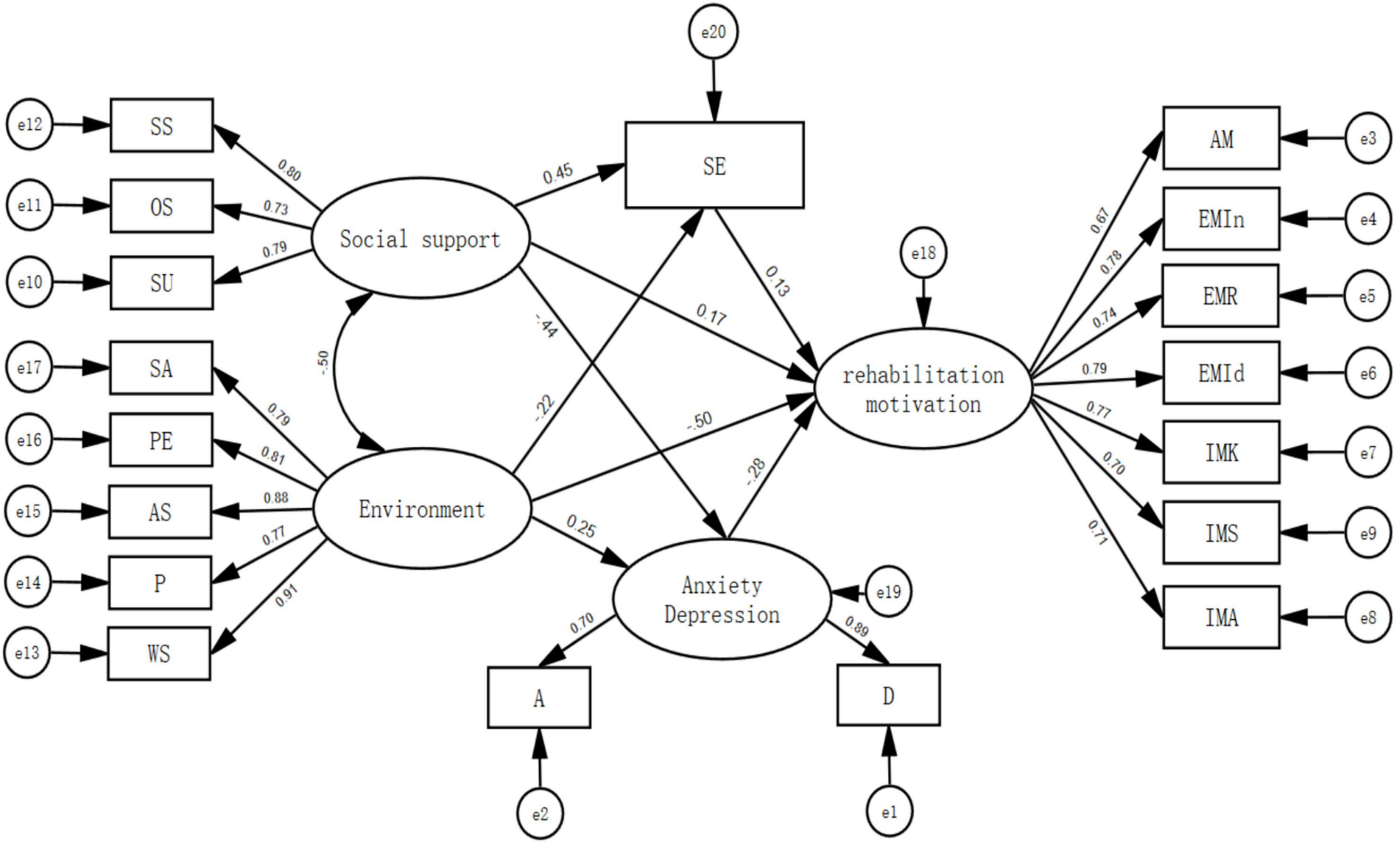
Structural equation model with standardized coefficients. SS: subjective support; OS: objective support; SU: support utilization; SA: services and assistance; PE: physical environment; AS: attitudes and support; P: policies; WS: work and study; SE: self-efficacy; A: anxiety; D: depression; AM: amotivation; EMIn: extrinsic motivation introjected; EMR: extrinsic motivation regulation; EMId: extrinsic motivation identification; IMK: intrinsic motivation knowledge; IMS: intrinsic motivation stimulation; IMA: intrinsic motivation accomplishment.

The revised model demonstrated good fit, with the following fit indices reported: *χ*^2^/df = 2.786, RMSEA = 0.065, NFI = 0.927, CFI = 0.952, TLI = 0.942, IFI = 0.952, and SRMR = 0.05. See Table 4 for details. The final structural equation model, which is shown in Figure 3. In this study, the standardized factor loadings for all variables ranged from 0.675 to 0.912, all reaching a significance level of 0.01. This indicates that each latent variable possesses strong explanatory power for the observed variables, all latent variables had composite reliability (CR) values between 0.78 and 0.92, all of which were higher than the suggested cutoff. Regarding convergent validity, all variables had average variance extracted (AVE) values between 0.53 and 0.70, all of which were higher than the recognized cutoff point of 0.5. As a result, this study’s measuring model exhibits strong convergent validity and reliability.

**Table 4:** Revised model inspection.

Fit indices	Recommended threshold	Scores	Remarks
*x*^2^/df	<5	2.786	Acceptable
NFI	>0.9	0.927	Acceptable
TLI	>0.9	0.942	Acceptable
IFI	>0.9	0.952	Acceptable
CFI	>0.9	0.952	Acceptable
RMSEA	<0.08	0.065	Acceptable
SRMR	<0.08	0.05	Acceptable

*x^2^/df:* Chi-square to degrees of freedom ratio; NFI: normed fit index; TLI: Tucker–Lewis index; IFI: incremental fit index; CFI: comparative fit index; RMSEA: root mean square error of approximation; SRMR: standardized root mean square residual.

#### 3.4.2 Testing the significance of the mediation pathway

According to the model results, through two paths, social support showed beneficial indirect relationships with rehabilitation motivation. First, there was a substantial indirect relationship through self-efficacy (standardized indirect effect = 0.059, 95% CI[0.01, 0.133]). Secondly, there was a significant indirect relationship through anxiety and depression (standardized indirect effect = 0.126, 95% CI[0.057, 0.244]). Through both mediators, environmental factors demonstrated unfavorable indirect relationships with rehabilitation motivation. Significant indirect relationships were found through self-efficacy (standardized indirect effect = −0.1, 95% CI[−0.08, −0.004]) and anxiety and depression (standardized indirect effect = −0.071, 95% CI[−0.165, −0.009]). See Table 5.

**Table 5:** Analysis of indirect effects in mediation models.

Path	Effect	Estimate	SE	95%*CI*		*p*
				Lower	Upper	
Social support → Rehabilitation motivation	Total effect	0.356	0.151	0.211	0.507	<0.001
	Direct effect	0.17	0.075	0.032	0.323	0.018
	Total indirect effect	0.186	0.076	0.11	0.296	<0.001
	Social support → Self-efficacy → Rehabilitation motivation	0.059	0.031	0.01	0.133	0.021
	Social support → Anxiety and depression → Rehabilitation motivation	0.126	0.045	0.057	0.244	<0.001
Environmental factors → Rehabilitation motivation	Total effect	−0.596	0.117	−0.727	−0.433	<0.001
	Direct effect	−0.496	0.06	−0.616	−0.383	<0.001
	Total indirect effect	−0.1	0.057	−0.196	−0.02	0.015
	Environmental factors → Self-efficacy → Rehabilitation motivation	−0.029	0.018	−0.08	−0.004	0.022
	Environmental factors → Anxiety and depression → Rehabilitation motivation	−0.071	0.039	−0.165	−0.009	0.023

## 4 Discussion

According to the study’s findings, stroke patients’ rehabilitation motivation is at a moderate level, which is in line with the findings published by Lee et al. (22). The intrinsic motivation-achievement category had the highest values, indicating that patients prioritize functional advancement and self-actualization during therapy. This is consistent with self-determination theory’s central idea of “competence needs” (9). The internal motivation-stimulation dimension, however, had the lowest results. This is directly related to the monotonous, repetitive character of stroke rehabilitation exercise, which is frequently accompanied by discomfort and exhaustion. It highlights enduring problems with present rehabilitation programs, like overuse of repetition and low participation. To improve the therapeutic experience and maintain intrinsic motivation, it is advised that digital technologies, such as virtual reality and motion-sensing games, be incorporated into rehabilitation.

According to the univariate analysis results, married patients have considerably greater levels of rehabilitation motivation than single and divorced patients, this may be because married patients have more reliable social support. Higher levels of education are linked to higher levels of rehabilitation motivation because educational attainment may affect patients’ comprehension of rehabilitation knowledge and their sense of the long-term benefit of rehabilitation. Furthermore, employed patients showed comparatively higher levels of rehabilitation motivation, which may be related to their increased social and familial duties as well as their more pressing need for functional recovery, which translates into a stronger internal rehabilitation motivation. Conversely, retired patients prioritized maintaining their health more, which led to a proportionally lower rehabilitation motivation; this is in line with the results of Li et al. (23). Additionally, compared to patients whose rehabilitation duration was one month or more, those whose period was less than one month showed noticeably reduced rehabilitation motivation. One explanation for this could be that early on after a stroke, patients may feel depressed and anxious because of their physical limitations and incapacity to take care of themselves, which lowers their rehabilitation motivation. However, patients progressively move from psychological adaptation to active self-management through active psychological adjustment and as rehabilitation advances. Their confidence in their rehabilitation may be strengthened by gradual functional improvements, starting a positive feedback loop that keeps them motivated. This finding has also been supported by Olsen et al. (24) research.

The results of this study show that important sociodemographic factors impacting stroke patients’ rehabilitation motivation include economic conditions, family support, employment status, and recovery stage. First, financial conditions have a significant impact on rehabilitation motivation. Increased monthly household income facilitates access to resources and the continuation of rehabilitation programs by offering the necessary financial stability for rehabilitation training. As a result, patients’ financial worries are reduced, allowing them to concentrate more on their recovery. This is consistent with the findings of Seo et al. (25). Second, rehabilitative motivation is significantly facilitated by strong family ties. In addition to meeting patients’ emotional and sense of belonging requirements, family support reduces psychological stress and identity crises brought on by sickness, which increases the intrinsic drive for healing. This result is consistent with Moon’s research (26) and supports the Self-Determination Theory’s view that the satisfaction of psychological needs promotes the development of motivation. This implies that the supportive function of the family system should be given priority in rehabilitation therapies, encouraging a change from “patient-centered” to “family-centered” care approaches. This study discovered that longer rehabilitation times were associated with greater patient motivation. This contrasts with Yoshida’s findings (27), possibly because of survivor bias in the study’s sample—patients who are able to maintain their rehabilitation frequently have higher levels of foundational motivation. Concurrently, patients go from psychological adaptation to active self-management as their rehabilitation advances. Their faith in their rehabilitation may be further bolstered by phased functional improvements, resulting in a beneficial feedback cycle that maintains motivation.

An important finding of this study is that while each individual psychosocial factor showed moderate correlations with rehabilitation motivation, together they explained a substantial 70.4% of the variance. This pattern—where multiple moderately correlated variables collectively yield high explanatory power—is characteristic of complex, multidimensional constructs. It suggests that rehabilitation motivation is not dominated by any single factor but emerges from the interplay of individual psychological resources (self-efficacy), emotional states (anxiety, depression), social support systems, and environmental conditions. This finding provides empirical support for the biopsychosocial model and Self-Determination Theory, which posit that human motivation is shaped by the satisfaction of multiple basic psychological needs across different domains.

The findings of this study indicate that self-efficacy and social support show positive direct associations with rehabilitation motivation, while depression, anxiety, and environmental obstacles demonstrate negative direct associations with rehabilitation motivation. It is consistent with the key concepts of self-determination theory: both the sensation of competence represented by self-efficacy and the sense of belonging satisfied by social support are essential psychological requirements for generating and maintaining intrinsic motivation (28). High self-efficacy improves patients’ belief in their influence over their rehabilitation process, which directly encourages active engagement in recovery activities (29, 30). On one hand, emotional support and encouragement from friends and family greatly increase patients’ confidence in how well they can rehabilitate and persevere (22). On the other hand, anxiety and depression not only deplete patients’ psychological energy, resulting in avoidance behaviors and a decreased awareness of rewards, but they also directly undermine their sense of purpose in pursuing recovery; external environmental barriers limit the conversion of motivation into sustained action. In clinical practice, “internal concerns” (emotional problems) and “external challenges” (environmental obstacles) must be managed together, using a comprehensive approach that takes into account both the body and the mind. It might entail minimizing external barriers through family counseling and environmental changes, while managing emotions through cognitive behavioral therapy and mindfulness training.

The findings indicate that social support and environmental factors are associated with rehabilitation motivation both directly and indirectly through their relationships with self-efficacy and anxiety-depression. Higher social support is related to higher self-efficacy and lower anxiety-depression, which in turn are related to higher rehabilitation motivation. The indirect pathway through anxiety-depression accounts for a larger proportion of the total association between social support and motivation compared to the pathway through self-efficacy. This implies that enhancing social support is essential for emotional control, which in turn significantly improves motivation, given the high frequency of anxiety and depression among stroke patients (31). Therefore, efforts in rehabilitation interventions should concentrate on creating a multidimensional social support system that includes families, caregivers, and other patients, in addition to directly optimizing the physical environment and improving policy and service support. This strategy seeks to improve patients’ self-efficacy, reduce their negative emotions, and eventually promote rehabilitation motivation in a synergistic way.

## 5 Limitations

Several limitations of this study should be acknowledged. First, the sample exhibits inherent selection bias, as the majority of participants who completed the questionnaire resided in towns and were married (71.9%), possessing stable family and social support systems; a review of the data suggests that this cohort likely consisted predominantly of patients with mild stroke. Second, as this study did not employ standardized neurological assessment tools such as the NIHSS, it was unable to control for the impact of stroke severity on rehabilitation motivation. It is recommended that future research incorporate objective neurological function indicators. Third, the generalizability of our findings is geographically constrained, as the sample was recruited from only two hospitals in Northwest China. Given that the economic and healthcare conditions in this region lag behind those in the South, the sample may not reflect the characteristics of stroke patients in other geographic regions, healthcare institutions, or cultural contexts. Finally, the cross-sectional design precludes any causal inferences, making it challenging to capture dynamic shifts in rehabilitation motivation over time. Therefore, longitudinal studies are warranted to explore the contributing factors and to fully document the beneficial effects of rehabilitation motivation throughout the stroke recovery process.

## 6 Conclusion

Stroke patients’ rehabilitation motivation is a continuous psychological process that is impacted by a variety of interconnected factors. Integrated intervention strategies should be used in future clinical rehabilitation practice, with an emphasis on managing emotional distress and improving self-efficacy at the individual level and strengthening social support networks, and optimizing the physical and policy environments for rehabilitation at the environmental level. Additionally, using interesting and digital rehabilitation techniques might enhance the training process, systematically boosting and maintaining patients’ rehabilitation motivation. In the end, this strategy improves quality of life and facilitates functional recovery.

## Data Availability

The original contributions presented in the study are included in the article/Supplementary material, further inquiries can be directed to the corresponding author.
