# Supplementary material for: An analysis of factors and pathways related to rehabilitation motivation in stroke patients based on self-determination theory
Source: Front Neurol. 2026 Apr 21;17:1789949. doi: 10.3389/fneur.2026.1789949 (PMC13141854; doi:10.3389/fneur.2026.1789949)
Supplement: Supplementary file 1 [file Data_Sheet_1.pdf]

Supplementary material

Summary of Instruments and Psychometric Properties

Instrument	Construct Measured	Dimensions/Subscales	Number of Items	Scoring Range	Reliability (Chinese Version)
SRMS	Rehabilitation motivation in stroke patients	• Amotivation<br>•Extrinsic motivation identification<br>•Extrinsic motivation regulation<br>•Extrinsic motivation introjected<br>•Intrinsic motivation knowledge<br>•Intrinsic motivation stimulation<br>•Intrinsic motivation accomplishment	28	1-5 (Likert)<br>Low: 28-56<br>Medium: 57-111<br>High: 112-140<br>Higher scores = greater motivation	• Test-retest reliability = 0.922<br>• Cronbach's α = 0.851
GSES	Self-efficacy	Single dimension	10	1-4 (Likert)<br>Higher scores = higher self-efficacy	• Cronbach's α = 0.87<br>• Test-retest reliability = 0.83
SSRS	Social support	• Subjective support<br>• Objective support<br>• Support utilization	10	12-66<br>Low: ≤22<br>Medium: 23-44<br>High: 45-66	• Cronbach's α = 0.89-0.94<br>• Test-retest reliability = 0.92
HADS	Anxiety and depression	• Anxiety subscale<br>• Depression subscale	14<br>(7 per subscale<br>)	0-3 (Likert) per item<br>Subscale range: 0-21<br>Higher scores = more severe symptoms	• Total scale:<br>Cronbach's α = 0.807<br>• Anxiety subscale:<br>Cronbach's α = 0.904<br>• Depression subscale:<br>Cronbach's α = 0.869
CHIEF	Environmental barriers	• Physical environment<br>• Attitudes / support<br>• Work / study<br>• Services /assistance<br>• Policies	12	Frequency (0-4) ×<br>Severity (0-2) per item<br>Higher scores = greater perceived environmental barriers	• Cronbach's α = 0.889<br>• Test-retest reliability = 0.800

SRMS = Stroke Rehabilitation Motivation Scale; GSES = General Self-Efficacy Scale; SSRS = Social Support Rating Scale; HADS = Hospital Anxiety and Depression Scale; CHIEF = Craig Hospital Inventory of Environmental Factors. All reliability coefficients reported are from Chinese versions of the instruments. All scales have been validated and applied in a sample of stroke patients and demonstrate sound psychometric properties.
